# Efficacy of Galvanism in Cases of Hydrophobia

**Published:** 1804-09-01

**Authors:** 


### Efficacy of Galvanism in Cases of Hydrophobia.

SOME experiments have been made at Turin, which prove the power of galvanism in the treatment of hydrophobia.

On the 19th of June, 1802, a man, aged 45 years, was bitten on the thumb of the right hand by his own dog, which was mad. On the 20th of August following, he went to consult professor Rosssi, as he had been seized with terror at the sight of water, which had produced convulsive movements in the muscles of the lower jaw. The The patient informed M. Rossi, that, during the first twenty-five days he had felt no pain, excepting in the part bitten; that he had, in consequence, cauterized it himself several times with boiling oil and the actual cautery, in order to excite a long suppuration; that on the 14th of July he felt a very acute pain in his neck, from which he was relieved by some internal composing medicines, which a professional man had prescribed; that, some days afterwards, being siezed with such a violent dizziness as to make him him fall down, an emetic was administered, which gave him very great relief; that, in a few days, he was attacked with pains in most of his joints, but particularly in the vertebræ of the neck and back; that finally, different remedies had been prescribed without success, till the day when his fear of the water had made him resolve to consult him.

M. Rossi having reason to believe, that madness would soon manifest itself in this man, as he already felt its symptoms, resolved, without loss of time, to have recourse to galvanism. He composed a pile of fifty couple, with disks of pasteboard wetted with a solution of muriate of ammonia. He established the mediate circle between the end of the thumb bitten and the commencement of the spinal marrow, and then between the tongue and the lower extremity of the spine; but the patient could not endure the operation on his tongue, without being excited to bite. He then placed him barefoot on the ground, which had been wetted with water, but he could not bear the impression made by that fluid. He was then removed to another place, and by means of a long conductor, which was carried from the end of his toes to his mouth, the galvanisation was continued only two minutes, because he threatened to bite. M. Rossi then fixed the immediate circle along the spinal marrow, and repeated the galvanisation till the patient fell into a deep swoon, which was succeeded by a profuse perspiration. A quarter of an hour afterwards the patient rose and was able to go home, accompanied by some friends, who were charged to give an account the following day, of what should happen in the interim.

The day following he returned by himself, saying that he was cured; for he had drank water with but a very slight degree of terror, and had taken a cup of chocolate. The galvanic operations were, on that day, repeated. He appeared appeared again on the fourth day, declaring that he experienced no difficulty either in eating or drinking, and refused to be galvanized. M. Rossi would not insist on performing the operation, on account of the excessive dread of galvanism testified by the patient; he only invited him to return the following day, but which, he however neglected to do till after an interval of six days. He then related, that, the preceding night, he had been seized with convulsive movements, and had been disturbed by frightful dreams. He likewise mentioned, that he frequently had an inclination to bite his clothes, and that he experienced great difficulty in drinking. He again submitted to the galvanic operation, which was performed in the manner above described, in the presence of M. Vassali Eandi, and several other persons. No symptom appeared from that time till the 14th day afterwards. During this interval, the patient came every day, of his own accord, to be galvanized, and received pleasure from it equal to the aversion he had before testified. On the fourteenth day he complained of pains in his joints, but M. Rossi perceiving that they were caused by the excessive use of galvanism, desisted from all the operations, and on the twenty-seventh day the pains went away. A few days afterwards, the patient again desired to be galvanized: his request was complied with, and, on the third day, the same pains returned; the operations were then suspended. Since that time, the patient has never experienced any symptom of his former complaint, and has enjoyed an excellent state of health.

The second observation was made on a hair dresser, who was bitten by a mad dog on the chin and the right leg, and who was sent to M. Rossi by M. Charron, Commissary General of Police, the fourth day after the accident, to be admitted into St. John's Hospital. The patient having employed no kind of remedy, M. Rossi thought it advisable to subject him to the ordinary treatment. He was therefore cauterized with the actual cautery on the two places where he had been bitten, and for twenty-six days after his admission into the hospital no other remedy was employed. Thirty-one days after the bite, the patient having complained that he could not sleep, opium was administered, but, instead of composing, it seemed only to increase his inquietude and agitation. The dose was increased to two grains, but without producing any benefit, and the patient even began to feel an obstruction in his throat. M. Rossi then proceeded to galvanise him, in the manner described in the preceding case. The galvanisation was repeated

 every every second day till the forty-sixth, when the patient appeared to be perfectly well. He left the hospital on the fifty-fourth day, and since that time has had no relapse.

